# The Combination of Atrial Fibrillation and Occlusion Site Predicts In Situ Atherosclerotic Thrombosis in Basilar Artery Occlusion

**DOI:** 10.3390/jcm14186384

**Published:** 2025-09-10

**Authors:** Yukishige Hashimoto, Fusao Ikawa, Reo Kawano, Toshikazu Hidaka, Yusuke Inoue, Yusuke Yamamoto, Nobutaka Horie

**Affiliations:** 1 Department of Neurosurgery, Shimane Prefectural Hospital, Izumo 693-0068, Japan; yukishigetotennis@gmail.com (Y.H.); thidaka55@yahoo.co.jp (T.H.); yusukeinoue0610@outlook.jp (Y.I.); 7852yy3215@gmail.com (Y.Y.); 2 Innovation Center for Translational Research, National Center for Geriatrics and Gerontology, Obu 474-8511, Japan; rkawano@ncgg.go.jp; 3 Department of Neurosurgery, Graduate School of Biomedical and Health Sciences, Hiroshima University, Hiroshima 734-8553, Japan; horie@hiroshima-u.ac.jp

**Keywords:** atrial fibrillation, basilar artery occlusion, cardioembolic, in situ atherosclerotic thrombosis, occlusion site, proximal occlusion

## Abstract

**Background/Objectives**: The accurate evaluation of stroke etiology in basilar artery occlusion (BAO) remains underexplored. This study aimed to explore a simple and practical method for predicting the in situ atherosclerotic thrombosis (ISAT) subtype of BAO. **Methods:** We retrospectively analyzed patients diagnosed with BAO at our institution between April 2015 and April 2025. ISAT was characterized by moderate-to-severe stenosis (>50%) at the occlusion site on angiography, while the cardioembolism (CE) subtype was defined as sudden-onset arterial occlusion with evidence of a cardiac source of embolism. The location of BAO was classified based on cerebral angiography findings as proximal, middle, or distal. Clinical and imaging characteristics were compared between CE and ISAT subtypes. **Results:** Among 33 patients, 8 (24%) were classified as having the ISAT subtype. Multivariable analyses revealed that the presence of atrial fibrillation (AF) (OR 0.03; 95% CI, 0.00–0.56) and a non-proximal occlusion site (i.e., middle or distal) (OR 0.02; 95% CI, 0.00–0.27) were independently associated with ISAT. Patients were stratified into four groups based on the presence or absence of proximal occlusion and AF. CE subtypes predominated in groups with either AF or no proximal occlusion (25/27, 93%), whereas ISAT was present in all patients with both proximal occlusion and absence of AF (6/6, 100%). The area under the curve for this classification was 0.955. The sensitivity, specificity, positive predictive value, and negative predictive value for diagnosing ISAT were 75%, 100%, 100%, and 93%, respectively. **Conclusions:** A simple classification based on the presence of proximal occlusion and AF status suggested the potential to facilitate preprocedural differentiation of ISAT subtype in BAO.

## 1. Introduction

Acute basilar-artery occlusion (BAO) accounts for approximately 1% of all ischemic strokes and 5% to 8% of large vessel occlusions (LVO) [1]. The basilar artery, formed by the confluence of the vertebral arteries, runs along the ventral brainstem and gives rise to major branches such as the paramedian perforators, anterior inferior cerebellar artery (AICA), superior cerebellar artery (SCA), and posterior cerebral arteries (PCA). Occlusions may occur at proximal, middle, or distal segments, with clinical manifestations varying accordingly: proximal occlusions often involve pontine perforators and cause severe brainstem syndromes with quadriplegia or coma, whereas distal occlusions more frequently affect the thalamus and occipital lobes, leading to altered consciousness, visual field deficits, or cognitive dysfunction. BAO represents one of the most devastating forms of stroke, with up to 80% of patients experiencing severe disability or death [2,3]. Two randomized controlled trials have demonstrated that endovascular treatment (EVT) for patients with BAO significantly reduces disability compared with best medical management alone [4,5]. Nonetheless, patients with in situ atherosclerotic thrombosis (ISAT) and cardioembolic (CE) subtypes may require distinct treatment approaches [6,7,8,9]. Although initial recanalization rates are comparable between ISAT and CE subtypes following EVT, re-occlusion is more common in ISAT, often requiring adjunctive procedures such as percutaneous transluminal angioplasty (PTA) or stenting along with antiplatelet therapy [10,11]. Therefore, accurately distinguishing ISAT from CE subtypes prior to EVT is essential for optimizing therapeutic strategies.

Recent studies have investigated various factors associated with the underlying etiology of BAO. Reported indicators suggestive of ISAT include the absence of atrial fibrillation (AF), a history of hypertension (HT), lower National Institutes of Health Stroke Scale (NIHSS) scores on admission, and proximal BAO [6,7,8,9,12,13,14,15]. Several diagnostic models have been developed for ISAT in anterior circulation LVO strokes, integrating clinical and imaging parameters, and have demonstrated high diagnostic accuracy [16,17]. However, diagnostic models for ISAT in BAO remain limited. To date, only one such model has been proposed; however, it lacks imaging-based variables and demonstrates inferior diagnostic performance compared to models used for anterior circulation stroke [18]. Therefore, the present study aimed to explore a simple and practical approach for predicting ISAT in BAO by utilizing readily accessible clinical and imaging data in the acute care setting.

## 2. Materials and Methods

### 2.1. Patient Population

This study was approved by the Institutional Review Board of Shimane Prefectural Central Hospital (R22-010; 12 July 2022). This ambispective, non-interventional study used an IRB-approved opt-out approach for both study arms, and written informed consent was waived; data were analyzed after de-identification. We performed a retrospective analysis of an ambispective cohort of consecutive patients who underwent EVT between April 2015 and April 2025; cases treated after 12 July 2022 were prospectively accrued under the approved protocol. Inclusion criteria were as follows: (1) the presence of acute neurological symptoms attributable to BAO, confirmed by computed tomography angiography (CTA) or magnetic resonance angiography (MRA); (2) presentation within 24 h of the estimated time of BAO onset; and (3) no evidence of intracranial hemorrhage on initial computed tomography (CT). Exclusion criteria included: (1) BAO due to embolism secondary to tandem vertebral artery steno-occlusion; (2) undetermined stroke etiology; or (3) other causes such as arterial dissection, vasculitis, or moyamoya disease.

### 2.2. Data Collection

Clinical data included demographic characteristics (age and sex); vascular risk factors such as HT, diabetes mellitus (DM), hyperlipidemia, AF—defined as either a history of AF or AF detected on physical examination or electrocardiography at admission—and smoking status, defined as either current or past smoking at the time of admission. Clinical manifestations were also recorded, including the NIHSS score, the presence of sudden onset, symptom progression, and coma on admission. Sudden onset was defined as the abrupt development of focal neurological symptoms that were either directly witnessed or clearly reported by the patient. Symptom progression was defined as a gradual worsening of neurological deficits prior to admission. Coma on admission was defined as a Glasgow Coma Scale score of ≤8, indicating a state of unresponsiveness requiring significant stimulation to elicit any purposeful response. Additional variables included the etiology of BAO, and imaging findings, such as the posterior circulation Alberta Stroke Program Early CT Score (pc-ASPECTS) [19], the presence of the hyperdense artery sign (HAS) [20], and the location of BAO. HAS was visually identified on baseline axial non-contrast CT scans with a section thickness of 5 mm and an interval of 5 mm. BAO location was classified based on cerebral angiography findings as follows: proximal (from the vertebrobasilar junction to the origin of the AICA), middle (from the origin of the AICA to the origin of the SCA), or distal (beyond the origin of the SCA) [13,21].

### 2.3. Classification of the Stroke Mechanism

Stroke etiology was determined based on clinical presentation, laboratory findings, and imaging characteristics, and was classified into ISAT and CE subtypes according to the TOAST (Trial of ORG 10172 in Acute Stroke Treatment) criteria [22]. All patients underwent routine evaluations to identify the underlying stroke mechanism, including transthoracic or transesophageal echocardiography, continuous electrocardiographic monitoring in a stroke unit or Holter monitoring, and cardiac CT. Patients suspected of having ISAT underwent additional vascular imaging after admission, including CTA, MRA, or DSA. The ISAT subtype was defined as in situ thrombosis in the basilar artery, characterized by moderate-to-severe stenosis (>50%) at the site of BAO on angiography, with or without a tendency for re-occlusion following recanalization. The CE subtype was defined as sudden-onset arterial occlusion with evidence of a cardiac embolic source, such as AF, valvular heart disease, prosthetic mitral or aortic valve, infective endocarditis, atrial or ventricular thrombus detected by echocardiography, cardiomyopathy, or structural cardiac anomalies, in the absence of BA stenosis.

### 2.4. Statistical Analysis

Categorical variables were presented as counts and percentages, while continuous variables were presented as means ± standard deviations. Comparisons between the ISAT and CE subtypes were performed using Fisher’s exact test for categorical variables and the Mann–Whitney U test for continuous variables. To facilitate analysis, we also considered simplifying the occlusion site classification from three categories (distal, middle, and proximal) to two categories, although the specific grouping criteria were determined in the analysis phase. Univariable and multivariable logistic regression analyses were performed to identify independent predictors of the ISAT subtype. Variables with a *p*-value < 0.05 in the univariable analysis were included in the multivariable model. Firth’s bias-reduced logistic regression was employed for variables exhibiting complete separation to ensure model convergence and reduce estimation bias. Adjusted odds ratios (ORs) and 95% confidence intervals (CIs) were reported. Receiver operating characteristic (ROC) curves were constructed to evaluate the diagnostic performance of relevant variables, with the false-positive rate (1—specificity) plotted on the x-axis and the true-positive rate (sensitivity) on the y-axis. All statistical analyses were conducted using JMP software (version 16; SAS Institute Inc., Cary, NC, USA). A two-tailed *p*-value of <0.05 was considered statistically significant.

## 3. Results

**Figure 1:**
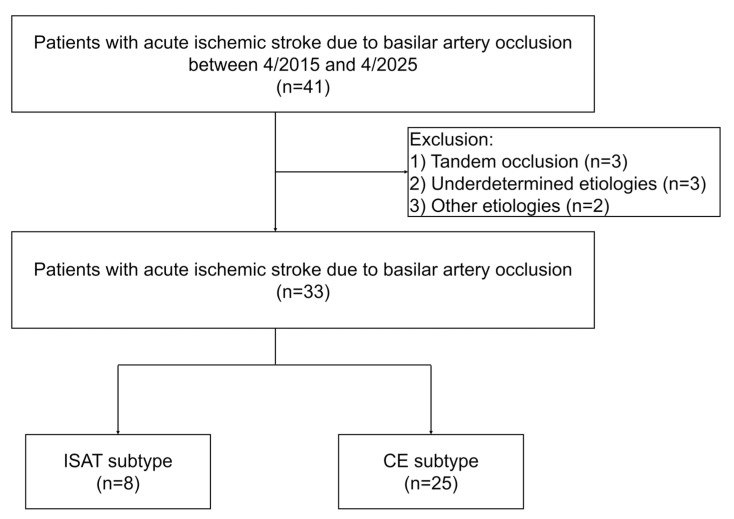
**Flowchart of patient selection.** A total of 41 patients with acute basilar artery occlusion (BAO) who underwent endovascular treatment were initially screened. Eight patients were excluded: three with embolism secondary to tandem vertebral artery steno-occlusion, three with undetermined etiology, and two with other causes, including arterial dissection (n = 2). The final study cohort consisted of 33 patients, classified as having either in situ atherosclerotic thrombosis (ISAT, n = 8) or cardioembolic (CE, n = 25).

Among 41 patients with acute BAO who underwent EVT between April 2015 and April 2025, 8 were excluded: 3 due to embolism secondary to tandem vertebral artery steno-occlusion, 3 with undetermined etiology, and 2 due to other causes, including arterial dissection (n = 2). A total of 33 patients were included in the present study (mean age, 82 ± 8.9 years; 21 men), as illustrated in the patient inclusion flowchart (Figure 1). The underlying stroke etiology was CE in 25 patients (76%) and ISAT in 8 patients (24%). Information regarding the presence or absence of a sudden onset was available in only 17 out of 33 patients (52%). Given this limited number, we excluded this variable from the comparative analysis to avoid potential bias. The mean NIHSS score at admission was 23 (range, 1–41), and the mean baseline pc-ASPECTS was 7 (range, 3–9). The locations of BAO were classified as proximal in 7 patients (21%), middle in 11 (33%), and distal in 15 (46%). Interrater agreement for the assessment of BAO etiology and occlusion site was substantial, with κ values of 0.76 (95% CI, 0.51–1.00; *p* < 0.0001) and 0.91 (95% CI, 0.78–1.00; *p* < 0.0001), respectively.

### 3.1. Baseline Characteristics

The baseline clinical characteristics and imaging findings are summarized in Table 1. Compared with the CE subtype, the ISAT subtype exhibited a lower NIHSS score at admission (14 ± 9.8 vs. 26 ± 13, *p* = 0.033). In addition, the distribution of BAO sites differed significantly between the two subtypes. ISAT was more frequently observed in proximal occlusions than in middle or distal occlusions. Conversely, CE was more frequently observed in distal than in middle and proximal occlusions (15/15 [100%] vs. 9/11 [82%] vs. 1/7 [14%], *p* < 0.001). Given the marked differences in subtype distribution across occlusion sites, we evaluated two binary classification schemes to simplify the three-tier categorization. When dichotomized by the presence or absence of distal occlusion, none of the patients with distal occlusion (0/15, 0%) were classified as having ISAT, whereas 8 of 18 patients (44%) without distal occlusion (i.e., middle or proximal occlusion) were classified as ISAT. Alternatively, when classification was based on the presence or absence of proximal occlusion, 6 of 7 patients (86%) with proximal occlusion were diagnosed with ISAT, compared with only 2 of 26 patients (7.7%) without proximal occlusion (i.e., middle or distal occlusion). Notably, classification based on the presence of proximal occlusion achieved higher diagnostic accuracy for identifying atherosclerotic etiology, as reflected by a greater area under the curve (AUC: 0.855 vs. 0.800).

### 3.2. Univariable and Multivariable Analyses of Potential Factors for BAO Due to ISAT

Univariable analyses demonstrated that the presence of AF (OR 0.03; 95% CI, 0.00–0.32) and a non-proximal occlusion site (i.e., middle or distal) (OR 0.02; 95% CI, 0.00–0.17) were significantly associated with a lower likelihood of ISAT (Table 2). In multivariable logistic regression analysis, the presence of AF and a non-proximal occlusion site were independently associated with a significantly lower likelihood of ISAT, with odds ratios of 0.03 (95% CI, 0.00–0.56) and 0.02 (95% CI, 0.00–0.27), respectively. ROC curve analysis demonstrated an AUC of 0.820 (95% CI, 0.724–0.916) for the absence of AF and 0.855 (95% CI, 0.690–1.000) for the presence of proximal occlusion in identifying ISAT subtypes (Table 3).

### 3.3. Classifications Based on the Combination of AF Status and the Presence of Proximal Occlusion

**Figure 2:**
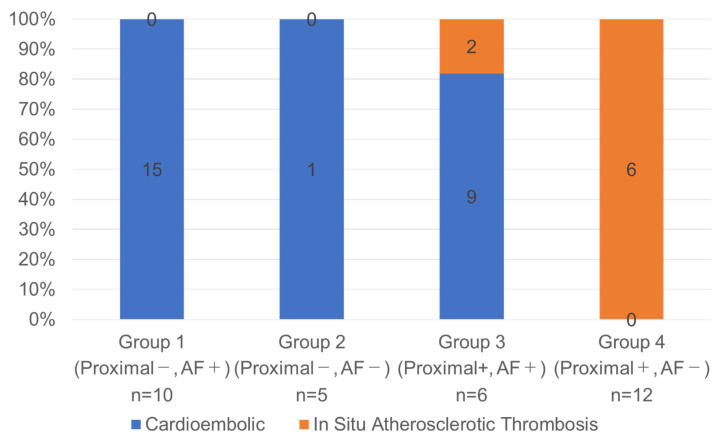
Stroke etiology distribution in basilar artery occlusion according to the four-group classification based on the combination of proximal occlusion and atrial fibrillation status. Patients with basilar artery occlusion (BAO) were stratified into four groups according to the presence or absence of proximal occlusion and atrial fibrillation (AF): Group 1 = proximal (−)/AF (+); Group 2 = proximal (−)/AF (−); Group 3 = proximal (+)/AF (+); Group 4 = proximal (+)/AF (−). Bar height indicates the proportion (%) of each stroke subtype (cardioembolic [CE] or in situ atherosclerotic thrombosis [ISAT]) within each group. The numbers within the bars represent absolute patient counts, and the total sample size for each group (n) is shown below the group labels.

To further assess the diagnostic value of combining clinical and imaging features, patients were stratified into four groups based on AF status and the presence of proximal occlusion: (1) proximal occlusion (−)/AF (+), (2) proximal occlusion (−)/AF (−), (3) proximal occlusion (+)/AF (+), and (4) proximal occlusion (+)/AF (−). CE strokes predominated in Groups 1–3 (25/27, 93%), whereas Group 4 consisted exclusively of ISAT cases (6/6, 100%), indicating a strong association (*p* < 0.001) (Figure 2).

**Figure 3:**
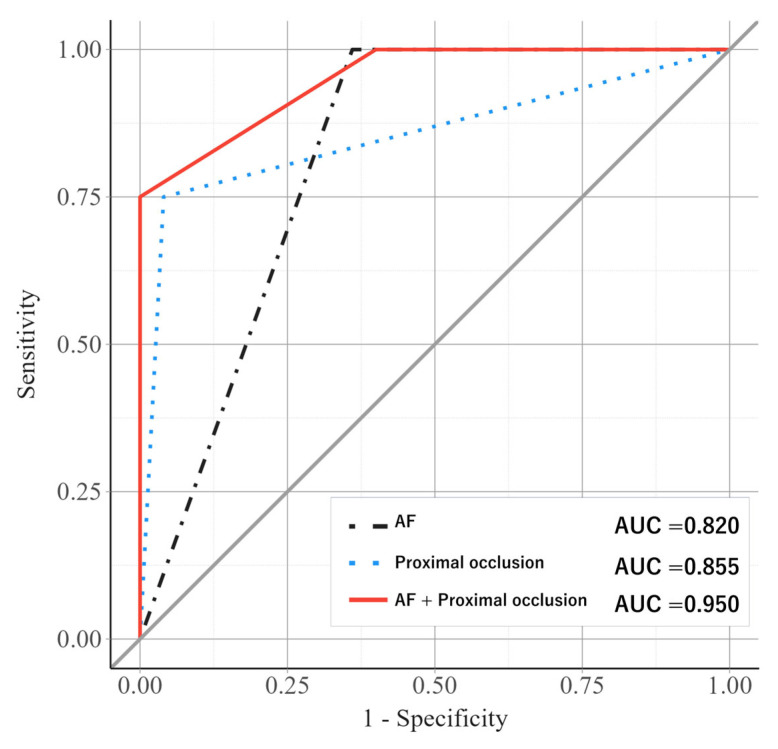
ROC curves for predicting in situ atherosclerotic thrombosis based on atrial fibrillation, proximal occlusion, and their combination. The predictive performance of atrial fibrillation (AF) alone (black dashed line), proximal occlusion alone (blue dotted line), and the combination of AF and proximal occlusion (red solid line) for identifying in situ atherosclerotic thrombosis in basilar artery occlusion. The gray diagonal line denotes the chance line, which reflects the performance of a non-informative test. A model with an AUC of 0.5 would fall along this line. The areas under the curve (AUCs) of AF alone, proximal occlusion alone, and AF + proximal occlusion were 0.820 (95% CI, 0.724–0.916), 0.855 (95% CI, 0.690–1.000), and 0.950 (95% CI, 0.881–1.000). The combination model demonstrated improved diagnostic accuracy compared to either variable alone (AF + proximal occlusion vs. AF, *p* = 0.009; AF + proximal occlusion vs. proximal occlusion, *p* = 0.07).

The AUC for this four-group classification model was 0.950 (95% CI, 0.881–1.000), demonstrating improved diagnostic accuracy compared to the use of either variable alone (AF + proximal occlusion vs. AF alone, *p* = 0.009; AF + proximal occlusion vs. proximal occlusion alone, *p* = 0.07) (Figure 3). The sensitivity, specificity, positive predictive value (PPV), and negative predictive value (NPV) for identifying BAO due to ISAT were 75%, 100%, 100%, and 93%, respectively.

## 4. Discussion

This study demonstrated that a simple classification combining the presence of proximal occlusion and AF status provided diagnostic accuracy for identifying atherosclerotic etiology than either variable alone. Notably, patients with both proximal occlusion and absence of AF exhibited a markedly high prevalence of ISAT, whereas those with either distal occlusion, AF, or both were rarely diagnosed with ISAT. These findings suggest that this readily available classification scheme may serve as a practical tool for the early identification of atherosclerotic BAO.

Previous studies have reported associations between stroke etiology in BAO and the site of occlusion [7,9,13,15]. We initially applied a three-tier classification of occlusion site (distal, middle, and proximal). However, to improve clinical applicability and simplify diagnostic decision-making, we reclassified the occlusion sites into two categories. When categorized by the presence of distal occlusion, all patients with distal occlusion (15/15, 100%) were diagnosed with the CE subtype (Table 1). Although this finding aligns with the conventional understanding that distal occlusion suggests a cardioembolic source, prior studies have reported that 9–27% of distal occlusions are attributable to underlying atherosclerosis [7,8,13,15]. This discrepancy highlights the risk of misclassification when relying solely on distal occlusion as an etiological indicator. In contrast, classification based on the presence of proximal occlusion demonstrated superior diagnostic performance in identifying atherosclerotic cases. In our cohort, 6 of 7 patients (86%) with proximal occlusion were classified as having ISAT, consistent with previous reports [7,8,13,15]. Given that the primary objective of this study was to identify ISAT cases requiring adjunctive therapies, we prioritized a proximal-based dichotomization. Notably, the diagnostic accuracy for predicting ISAT was higher when using proximal occlusion as a classifier compared to distal occlusion (AUC: 0.855 vs. 0.800), further supporting the validity of this classification approach.

Univariable and multivariable analyses using Firth’s bias-reduced logistic regression revealed that AF status and occlusion site were independently associated with the ISAT subtype (Table 2), consistent with previous reports [7,8,13]. Specifically, the absence of AF showed high sensitivity, while proximal occlusion demonstrated high specificity and PPV (Table 3). When used in combination, these complementary markers significantly enhanced diagnostic accuracy compared to either variable alone. Patients with AF or a non-proximal occlusion were predominantly classified as having CE subtype, whereas those with both absence of AF and proximal occlusion were highly likely to have ISAT. These findings suggest that a simple classification model incorporating AF and the presence of proximal occlusion may facilitate accurate differentiation of stroke subtypes in BAO prior to treatment and support the selection of appropriate therapeutic strategies.

The present study demonstrated that proximal occlusion was associated with ISAT subtype, whereas distal occlusion was associated with the CE subtype. The underlying mechanisms linking stroke etiology and occlusion site may be explained from the perspective of the pathophysiological mechanisms. In the proximal basilar artery, particularly at the vertebrobasilar confluence, the vascular geometry produces low and oscillatory wall shear stress, which promotes endothelial dysfunction and localized atherogenesis [23,24,25]. This hemodynamic phenomenon has been consistently observed in both computational fluid dynamics simulations and histopathological studies. Furthermore, recent vessel wall MRI studies have correlated these unstable hemodynamic conditions with high-risk plaque features in this region, which is composed of intraplaque hemorrhage [26,27]. Plaque rupture or endothelial erosion then initiates platelet aggregation and local thrombosis, resulting in vessel occlusion at the site of the lesion. Because this process is rooted in chronic vascular pathology, ISAT is more strongly associated with proximal BAO and often exhibits resistance to conventional thrombectomy. In contrast, thrombi in AF-related CE subtypes typically originate within the atrial appendage due to blood stasis, endothelial dysfunction, and a prothrombotic state associated with atrial remodeling. These thrombi are usually large and friable, and once dislodged, they migrate along the arterial tree until they lodge in narrower segments, most often at distal or bifurcation sites of the basilar artery [15,21]. This mechanism explains the predominance of CE events in distal BAO.

The present study proposed a practical yet informative classification model that incorporates two readily available clinical parameters: AF status and the presence of proximal occlusion. Both parameters can be rapidly assessed in emergency settings without the need for advanced imaging or specialized interpretation, thereby enhancing their clinical utility. Stratification into four subgroups based on these factors yielded high diagnostic accuracy in identifying the ISAT subtype. Notably, patients with both the absence of AF and the presence of proximal occlusion exhibited a particularly high likelihood of the ISAT subtype. This classification carries two major clinical implications. First, early recognition of the need for adjunctive interventions: Accurate preprocedural identification of ISAT allows clinicians to anticipate that conventional thrombectomy alone may be insufficient and that adjunctive therapies, such as percutaneous transluminal angioplasty or stenting combined with antiplatelet therapy, will likely be required [9,11]. Recent studies have demonstrated that early aggressive stenting for ISAT-related BAO is associated with improved functional outcomes without an increased risk of hemorrhagic complications [7,8,13,14]. Moreover, when ISAT is strongly suspected preprocedurally, operators may avoid repeated stent retriever maneuvers at the stenotic site, thereby reducing the risk of plaque injury or distal embolization. Second, earlier initiation of antiplatelet therapy: In ISAT, the underlying stenotic plaque is often unstable, and recurrent thrombosis may occur even after clot retrieval. Early administration of antiplatelet agents, guided by accurate preprocedural diagnosis, may therefore be crucial for maintaining durable reperfusion. Furthermore, in patients strongly suspected of having an atherosclerotic etiology, it may be reasonable to avoid intravenous thrombolysis and instead prioritize antiplatelet therapy, potentially reducing the risk of hemorrhagic complications. Therefore, accurate preprocedural identification of ISAT enables timely preparation for adjunctive interventions and early antiplatelet administration, potentially reducing re-occlusion and improving overall clinical outcomes.

This study has several limitations. First, it was conducted at a single institution with a relatively small sample size, which may have limited the statistical power of the analysis. Although our data suggest a segment–etiology pattern—with distal occlusions more often CE and proximal occlusions more often ISAT—overlap between occlusion site and etiology cannot be excluded. More precise differentiation will likely require multivariable models that integrate clinical, laboratory, and imaging variables beyond occlusion site, with prospective validation in larger, multicenter cohorts. Second, all participants in this study were of Japanese ethnicity, which raises concerns regarding the applicability of our findings to other racial and geographic populations. External validation in ethnically and geographically diverse cohorts, including those in Europe and North America, will be essential to confirm the broader utility of this approach. Finally, sudden onset was not included as a variable in the analysis due to the small number of patients with reliable onset information (17 out of 33 cases), which may have limited our ability to fully evaluate the temporal characteristics of symptom onset. Despite these limitations, the present study proposes a diagnostic model with high accuracy for identifying ISAT in BAO, which may contribute meaningfully to future stratified treatment strategies.

## 5. Conclusions

A simple classification based on the presence of proximal occlusion and AF status suggested the potential to aid in the preprocedural differentiation of the ISAT subtype in BAO, which may support tailored EVT strategies and antithrombotic selection.

## Figures and Tables

**Table 1:** Comparisons of characteristics of in situ atherosclerotic thrombosis and cardioembolic subtypes in basilar artery occlusion.

Characteristics	ISAT (n = 8)	CE (n = 25)	*p* Value
Patient demographics			
Age (years)	78 ± 11	83 ± 7.9	0.35
Male	4 (50)	8 (32)	0.42
Risk factor			
Hypertension	8 (100)	18 (72)	0.15
Hyperlipidemia	5 (63)	13 (52)	0.70
Diabetes mellitus	2 (25)	13 (52)	0.24
Atrial fibrillation	0 (0)	16 (64)	**0.003**
Smoking history (past or current)	4 (50)	11 (44)	1.0
History of ischemic stroke	1 (13)	6 (24)	0.65
Clinical manifestation			
Symptom progression	4 (50)	5 (20)	0.17
Presence of coma on admission	2 (25)	14 (56)	0.22
Baseline NIHSS score	14 ± 9.8	26 ± 13	**0.033**
Radiological characteristics			
Occlusion site			**<0.001**
Proximal	6 (75)	1 (4)	
Middle	2 (25)	9 (36)	
Distal	0 (0)	15 (60)	
Hyperdense artery sign	5 (63)	22 (88)	0.14
pc-ASPECTS	6.4 ± 2.3	6.7 ± 1.7	1.0

Values are shown as numbers (%) or means ± standard deviations unless otherwise indicated. The boldface type indicates a significant difference (*p* < 0.05). CE, cardioembolism; ISAT, in situ atherosclerotic thrombosis; NIHSS, National Institutes of Health Stroke Scale; and pc-ASPECTS, posterior circulation-acute stroke prognosis early CT score.

**Table 2:** Univariate and multivariate logistic regression analyses of potential factors for basilar artery occlusion due to in situ atherosclerotic thrombosis.

	Univariate Analysis		Multivariate Analysis	
	OR (95% CI)	*p* Value	OR (95% CI)	*p* Value
Age	0.95 (0.86–1.03)	0.216		
Male	0.49 (0.10–2.32)	0.359		
Hypertension	6.89 (0.69–932.94)	0.112		
Diabetes mellitus	0.36 (0.06–1.71)	0.203		
Hyperlipidemia	1.46 (0.32–7.46)	0.632		
Atrial fibrillation	0.03 (0.00–0.32)	**0.001**	0.03 (0.00–0.56)	**0.014**
Smoking history	1.26 (0.27–5.96)	0.765		
Symptom progression	4.00 (0.73–21.8)	0.109		
Presence of coma on admission	0.26 (0.04–1.56)	0.141		
Baseline NIHSS score	0.94 (0.87–1.00)	0.067		
Hyperdense artery sign	0.24 (0.04–1.44)	0.117		
pc-ASPECTs	0.91 (0.60–1.40)	0.649		
Occlusion site (distal/middle)	0.02 (0.00–0.17)	**<0.001**	0.02 (0.00–0.27)	**0.001**

The boldface type indicates a significant difference (*p* < 0.05). NIHSS, National Institutes of Health Stroke Scale; pc-ASPECTS, posterior circulation-acute stroke prognosis early CT score.

**Table 3:** Diagnostic accuracy of potential factors for basilar artery occlusion due to in situ atherosclerotic thrombosis.

	Sensitivity, %	Specificity, %	PPV, %	NPV, %	AUC
Atrial fibrillation	100	64	47	100	0.820
Proximal occlusion	75	96	86	82	0.855

AUC indicates the area under the curve; NPV, negative predictive value; and PPV, positive predictive value.

## Data Availability

All data are available upon request from the corresponding author upon reasonable request.

## Abbreviations

The following abbreviations are used in this manuscript:

AF	atrial fibrillation
BAO	basilar-artery occlusion
CE	cardioembolic
CTA	computed tomography angiography
EVT	endovascular treatment
HAS	hyperdense artery sign
HT	hypertension
ISAT	in situ atherosclerotic thrombosis
LVO	large vessel occlusion
MRA	magnetic resonance angiography
NIHSS	National Institutes of Health Stroke Scale
pc-ASPECTS	posterior circulation Alberta Stroke Program Early CT Score

## References

[1] Smith W.S., Lev M.H., English J.D., Camargo E.C., Chou M., Johnston S.C., Gonzalez G., Schaefer P.W., Dillon W.P., Koroshetz W.J. (2009). Significance of large vessel intracranial occlusion causing acute ischemic stroke and TIA. Stroke.

[2] Lindsberg P.J., Sairanen T., Nagel S., Salonen O., Silvennoinen H., Strbian D. (2016). Recanalization treatments in basilar artery occlusion-Systematic analysis. Eur. Stroke J..

[3] Nguyen T.N., Strbian D. (2021). Endovascular Therapy for Stroke due to Basilar Artery Occlusion: A BASIC Challenge at BEST. Stroke.

[4] Jovin T.G., Li C., Wu L., Wu C., Chen J., Jiang C., Shi Z., Gao Z., Song C., Chen W. (2022). Trial of Thrombectomy 6 to 24 Hours after Stroke Due to Basilar-Artery Occlusion. N. Engl. J. Med..

[5] Tao C., Nogueira R.G., Zhu Y., Sun J., Han H., Yuan G., Wen C., Zhou P., Chen W., Zeng G. (2022). Trial of Endovascular Treatment of Acute Basilar-Artery Occlusion. N. Engl. J. Med..

[6] Sun X., Raynald, Tong X., Gao F., Deng Y., Ma G., Ma N., Mo D., Song L., Liu L. (2021). Analysis of Treatment Outcome After Endovascular Treatment in Different Pathological Subtypes of Basilar Artery Occlusion: A Single Center Experience. Transl. Stroke Res..

[7] Liu H., Zeng G., Zeng H., Yu Y., Yue F., Ke Y., Yan Z., Pu J., Zhang J., Wei W. (2022). Endovascular treatment for acute basilar artery occlusion due to different stroke etiologies of large artery atherosclerosis and cardioembolism. Eur. Stroke J..

[8] Mierzwa A.T., Al Kasab S., Nelson A., Gutierrez S.O., Vivanco-Suarez J., Farooqui M., Jadhav A.P., Desai S., Toth G., Alrohimi A. (2024). Thrombectomy Outcomes in Acute Basilar Artery Occlusions Due to Intracranial Atherosclerotic Disease. Neurosurgery.

[9] Yuan G., Nguyen T.N., Liu L., Li R., Xia H., Long C., Wu J., Xu J., Huang F., He B. (2024). Effect of Stroke Etiology on Endovascular Treatment for Acute Basilar-Artery Occlusion: A Post Hoc Analysis of the ATTENTION Randomized Trial. Stroke.

[10] Psychogios M., Brehm A., López-Cancio E., De Marchis G.M., Meseguer E., Katsanos A.H., Kremer C., Sporns P., Zedde M., Kobayashi A. (2022). European Stroke Organisation guidelines on treatment of patients with intracranial atherosclerotic disease. Eur. Stroke J..

[11] de Havenon A., Zaidat O.O., Amin-Hanjani S., Nguyen T.N., Bangad A., Abbasi M., Anadani M., Almallouhi E., Chatterjee R., Mazighi M. (2023). Large Vessel Occlusion Stroke due to Intracranial Atherosclerotic Disease: Identification, Medical and Interventional Treatment, and Outcomes. Stroke.

[12] Lee Y., Yoon W., Kim S., Baek B., Kim G., Kim J., Park M. (2017). Acute Basilar Artery Occlusion: Differences in Characteristics and Outcomes after Endovascular Therapy between Patients with and without Underlying Severe Atherosclerotic Stenosis. Am. J. Neuroradiol..

[13] Baik S.H., Park H.J., Kim J.H., Jang C.K., Kim B.M., Kim D.J. (2019). Mechanical Thrombectomy in Subtypes of Basilar Artery Occlusion: Relationship to Recanalization Rate and Clinical Outcome. Radiology.

[14] Yu W., Higashida R.T. (2022). Endovascular Thrombectomy for Acute Basilar Artery Occlusion: Latest Findings and Critical Thinking on Future Study Design. Transl. Stroke Res..

[15] Mutke M.A., Potreck A., Schmitt N., Seker F., Ringleb P.A., Nagel S., Möhlenbruch M.A., Bendszus M., Weyland C.S., Jesser J. (2023). Exact Basilar Artery Occlusion Location Indicates Stroke Etiology and Recanalization Success in Patients Eligible for Endovascular Stroke Treatment. Clin. Neuroradiol..

[16] Liao G., Zhang Z., Tung T.-H., He Y., Hu L., Zhang X., Chen H., Huang J., Du W., Li C. (2022). A simple score to predict atherosclerotic or embolic intracranial large-vessel occlusion stroke before endovascular treatment. J. Neurosurg..

[17] Chen W., Liu J., Yang L., Sun H., Yang S., Wang M., Qin W., Wang Y., Wang X., Hu W. (2024). Development and internal-external validation of the ATHE Scale: Predicting acute large vessel occlusion due to underlying intracranial atherosclerosis prior to endovascular treatment. J Neurosurg..

[18] Zha M., Wu M., Huang X., Zhang X., Huang K., Yang Q., Cai H., Ji Y., Lv Q., Yang D. (2021). A Pre-Interventional Scale to Predict in situ Atherosclerotic Thrombosis in Acute Vertebrobasilar Artery Occlusion Patients. Front. Neurol..

[19] Khatibi K., Nour M., Tateshima S., Jahan R., Duckwiler G., Saver J., Szeder V. (2019). Posterior Circulation Thrombectomy-pc-ASPECT Score Applied to Preintervention Magnetic Resonance Imaging Can Accurately Predict Functional Outcome. World Neurosurg..

[20] Liebeskind D.S., Sanossian N., Yong W.H., Starkman S., Tsang M.P., Moya A.L., Zheng D.D., Abolian A.M., Kim D., Ali L.K. (2011). CT and MRI early vessel signs reflect clot composition in acute stroke. Stroke.

[21] Cross D.T., Moran C.J., Akins P.T., Angtuaco E.E., Diringer M.N. (1997). Relationship between clot location and outcome after basilar artery thrombolysis. Am. J. Neuroradiol..

[22] Adams H.P., Bendixen B.H., Kappelle L.J., Biller J., Love B.B., Gordon D.L., Marsh E.E. (1993). Classification of subtype of acute ischemic stroke. Definitions for use in a multicenter clinical trial. TOAST. Trial of Org 10172 in Acute Stroke Treatment. Stroke.

[23] Wake-Buck A.K., Gatenby J.C., Gore J.C. (2012). Hemodynamic characteristics of the vertebrobasilar system analyzed using MRI-based models. PLoS ONE.

[24] Kim B.J., Lee K.M., Kim H.Y., Kim Y.S., Koh S.-H., Heo S.H., Chang D.-I. (2018). Basilar Artery Plaque and Pontine Infarction Location and Vascular Geometry. J. Stroke.

[25] Sun J., Liu G., Zhang D., Wu Z., Liu J., Wang W. (2021). The Longitudinal Distribution and Stability of Curved Basilar Artery Plaque: A Study Based on HR-MRI. J. Atheroscler. Thromb..

[26] Li S., Wei J., Huang R., Li C., Chen H., Qiu Z., Jiang Y., Wu L. (2022). High-risk features of basilar artery atherosclerotic plaque. Front. Neurol..

[27] Xiong J., Liu Y., Mei L., Zhang C., Xia J., Chen H., Qu X., Wu J. (2025). Vessel wall imaging of vertebrobasilar artery configurations associated with posterior circulation infarction and high-risk atherosclerotic plaques. Sci. Rep..

